# Fluorinated Porcine Bone-Derived Hydroxyapatite Promotes Vascularized Osteogenesis by Coordinating Human Bone Marrow Mesenchymal Stem Cell/Human Umbilical Vein Endothelial Cell Complexes

**DOI:** 10.3390/bioengineering11121287

**Published:** 2024-12-18

**Authors:** Xiayi Wu, Chunxin Xu, Junming Feng, Shiyu Wu, Runheng Liu, Wei Qiao, Xin Luo, Shoucheng Chen, Zhipeng Li, Zhuofan Chen

**Affiliations:** 1 Hospital of Stomatology, Guanghua School of Stomatology, Sun Yat-sen University and Guangdong Research Center for Dental and Cranial Rehabilitation and Material Engineering, Guangzhou 510055, China; wuxiayi@mail.sysu.edu.cn (X.W.); hsuchh@mail2.sysu.edu.cn (C.X.); fengjm9@mail2.sysu.edu.cn (J.F.); wushy55@mail.sysu.edu.cn (S.W.); liurh28@mail.sysu.edu.cn (R.L.); chenshch8@mail.sysu.edu.cn (S.C.); 2 Applied Oral Sciences and Community Dental Care, Faculty of Dentistry, The University of Hong Kong, Hong Kong, China; qiaowei@connect.hku.hk

**Keywords:** angiogenesis, biological hydroxyapatite, fluoride

## Abstract

Biogenic hydroxyapatite is known for its osteoinductive potential due to its similarity to human bone and biocompatibility, but insufficient vascularization compared to autogenous bone during early implantation limits bone integration and osteogenesis. Fluorine has been shown to improve hydroxyapatite’s mechanical properties and the coupling of osteogenic and angiogenic cells. In this study, fluorine-modified biogenic hydroxyapatite (FPHA) with varying fluorine concentrations was prepared and tested for its ability to promote vascularized osteogenesis. FPHA prepared in this study retained the natural porous structure of biological cancellous bone and released F^−^ ions when immersed in cell culture medium. The extraction solutions of FPHA0.25 and FPHA0.50 promoted the formation of capillary-like tubes by human umbilical vein endothelial cells (HUVECs), with FPHA0.25 significantly upregulating *vegf* mRNA and VEGF protein levels in co-cultured human bone marrow mesenchymal stem cells (HBMSCs). Additionally, FPHA0.25 and FPHA0.50 upregulated *pdgf-bb* mRNA and PDGF-BB protein levels in HUVECs. In vivo experiments using a rabbit cranial defect model demonstrated that FPHA0.25 promoted early bone formation and angiogenesis in the defect area, enhanced VEGF secretion, and increased PDGFR-β expression in endothelial and mesenchymal cells. These findings suggest that fluorine-modified biogenic hydroxyapatite with an optimal fluorine concentration (FPHA0.25) may offer a promising strategy to enhance the body’s innate bone-healing potential by accelerating vascularization.

## 1. Introduction

Hydroxyapatite (HA) is a calcium phosphate compound similar to the primary mineral component of human bone. It exhibits excellent biocompatibility, bioactivity, and osteoconductivity, making it widely used in bone repair [1,2]. The porous structure of HA provides a scaffold for bone formation and promotes bone regeneration through the adsorption and release of bioactive molecules [3]. Additionally, HA plays a crucial role in cartilage defect repair and osteoarthritis treatment by enhancing chondrocyte migration, adhesion, and proliferation, as well as regulating the secretion and maturation of the cartilage matrix [4,5,6]. In recent years, HA has been applied in bone graft materials, bone cements, and tissue engineering scaffolds to facilitate bone and cartilage repair and regeneration [7,8]. However, in the early stages following the in vivo implantation of biogenic hydroxyapatite, cells located beyond 200 μm from the scaffold edge often experience inadequate oxygen and nutrient supply [9]. This insufficient blood supply can compromise the bone integration of bone substitute materials and bone tissue engineering grafts in the host area, often resulting in limited osteogenesis around the grafting area periphery. Consequently, the speed, volume, and quality of bone formation are significantly constrained by insufficient blood supply, limiting the material’s clinical application [10]. Beyond delivering essential oxygen and nutrients for cell survival and removing metabolic waste, the vasculature also supplies growth factors and other pro-osteogenic substances that stimulate stem cell maturation, differentiation, and migration, thus enhancing bone tissue formation [11]. Mineral deposition, critical for bone matrix mineralization, similarly relies on the neovascular system. Thus, effective bone regeneration in defect areas requires a coordinated interplay between vascularization and osteogenesis, making vascular reconstruction within biogenic hydroxyapatite scaffolds essential to maximizing their regenerative potential [12].

Contemporary strategies to enhance vascularization in bone regeneration focus on two main areas: cellular guidance and subcellular-level biological modulation. Cellular guidance primarily involves incorporating active cultured cells (such as endothelial cells, smooth muscle cells, mesenchymal stem cells, or co-cultured combinations) onto material scaffolds ex vivo or locally releasing gene-modified cells prior to in vivo implantation to promote osteogenesis and vascularization [13,14]. Subcellular-level biological induction strategies include loading growth factors (BMP-2, VEGF, IGF, bFGF, etc.) and other cell signaling molecules (e.g., fibronectin, RGD, SDF-1) through methods such as heparin mediation, ion complexation, microsphere loading, or protein modification [15]. However, these strategies face challenges in terms of high costs, uncontrolled release stability, insufficient long-term efficacy, immune rejection, and safety concerns. Consequently, research attention has refocused on the intrinsic properties of biomaterials [16].

Fluorine, an essential trace element in humans, has been employed to enhance the crystallinity of hydroxyapatite, reduce its solubility, and improve its mechanical properties [17]. Simultaneously, fluoride ions have been shown to stimulate the coupling of osteogenic and angiogenic progenitor cells, a process crucial for vascularized osteogenesis. For instance, fluoride enhances the angiogenic potential of MC3T3-E1 cells, a murine embryonic osteoblast precursor cell [18]. Furthermore, studies have demonstrated that cobalt (Co^2+^)- and fluoride (F^−^)-co-doped calcium phosphate (CaP) ceramics promote the expression of angiogenic markers such as VEGF and CD31 in bone marrow-derived mesenchymal stem cells (BMSCs), while also enhancing osteogenic differentiation, suggesting the dual promotion of both osteogenesis and angiogenesis [19]. These findings highlight the angiogenic potential of fluoride-doped materials, positioning them as promising candidates for next-generation bone substitutes.

This study aims to investigate the mechanisms and effects of hydroxyapatite with gradient fluorine concentrations in promoting bone regeneration vascularization and facilitating early new bone formation through angiogenic–osteogenic coupling. The findings are expected to provide theoretical insights into the biological transformation process and molecular mechanisms of such materials, as well as to support their subsequent clinical translation. In this study, we prepared fluorine-modified biogenic hydroxyapatite with a gradient of fluorine concentrations and evaluated its capacity to promote vascularized osteogenesis by modulating the interactions between human bone marrow mesenchymal stem cells (HBMSCs) and human umbilical vein endothelial cells (HUVECs) in vitro. Additionally, we examined its in vivo effects on angiogenesis, osteogenesis, and the expression of key regulatory factors. Fluorine-modified biogenic hydroxyapatite may offer a promising strategy to enhance the body’s innate bone-healing potential by accelerating vascularization.

## 2. Materials and Methods

### 2.1. Preparation of Fluorinated Porcine Bone Substitutes

The preparation of FPHA consisted of two steps, as described in our previous study [20]. Briefly, PHA was prepared from porcine cancellous bone. Macroscopic impurities were first removed from the bone by boiling in an autoclave at 121 °C for 30 min. Then, calcination was carried out at 800 °C for 2 h in air (heating rate: 10 °C/min) in a muffle furnace (SGM6812BK, Sigma Furnace Industry, Shanghai, China). The thermally treated samples, known as PHA, were immersed in sodium fluoride aqueous solutions of varied fluoride concentrations (F^−^: 0.25, 0.50, and 0.75 mol·L^−1^) for 24 h. After chemical treatment in the sodium fluoride solution, calcination was carried out at 700 °C for 3 h in air (heating rate: 10 °C/min). Thermally treated samples, known as FPHA, were cooled down at room temperature, subsequently rinsed in sufficient deionized water three times to remove the excess sodium fluoride, and then dried at 80 °C for 12 h. Finally, all samples were ground and sieved into particles with a size of 250–1000 μm. All samples were sterilized and dehydrated before use.

Ionic extracts of FPHA were prepared as reported [21]. Granules (0.5 g) of PHA, FPHA0.25, FPHA0.50, and FPHA0.75 were soaked in 5 mL of serum-free α-MEM basic medium (Gibco, Life technologies, Shanghai, China) and incubated in a humidified atmosphere containing 5% CO_2_ for 24 h at 37 °C. The supernatant (extract) was collected and sterilized through a filter (MillexGP, 0.22 μm, Millipore, Merck Millipore, Tullagreen, Carrigtwohill, Ireland) and stored at 4 °C (ISO10993-1) [22] for further use.

Extracts of FPHA0.25, FPHA0.50, and FPHA0.75 were diluted with the same volume of ionic strength adjustment buffer solution (TISAB II with CDTA, Thermo Scientific, Chelmsfold, MA, USA) for evaluation. The fluoride ion concentration was determined with a fluoride-selective electrode (7102, Ruosull, Shanghai, China) connected to an ion analyzer (Orion DUAL STAR pH/ISE meter, Thermo Scientific, Singapore). Calibration was performed each time before testing according to the manufacturer’s instructions.

### 2.2. In Vitro Study

#### 2.2.1. Culture of HBMSCs and HUVECs

HBMSCs were obtained from Cyagen Biosciences Inc. (Guangzhou, China). HBMSCs were resuspended in D-MEM (Gibco, Life technologies, Shanghai, China) containing 10% (*v*/*v*) fetal bovine serum (FBS, Australia Origin, Gibco, Life technologies, Frederick, MD, USA) with 100 units/mL of penicillin and 100 μg/mL of streptomycin (Gibco, Thermo fisher, Waltham, MA, USA), and subcultured at a concentration of 2000 cells/cm^2^ in a humidified atmosphere containing 5% CO_2_ at 37 °C. The CD marker (CD90, CD73, CD14, and CD45, Biolegend, San Diego, CA, USA) expression of HBMSCs was identified by flow cytometry. Multipotent differentiating capacity (osteocytes, adipocytes, and chondrocytes) was confirmed by osteogenic, chondrogenic, and adipogenic inductions (Cyagen, Santa Clara, CA, USA) according to instructions. Alizarin red S, alizarin blue, and oil-red O staining for differentiated HBMSCs were performed.

HUVECs were a courtesy from a researcher Dr. Yujie Liu (Sun Yat-sen Memorial Hospital, Sun Yat-sen University). The cells were subsequently collected and grown in complete ECM (Sciencell, San Diego, CA, USA). The obtained cells were immunofluorescently stained with antibodies (Ab) against von Willebrand Factor (vWF, Pierce, Thermo scientific, Rockford, IL, USA) and Vascular Endothelial cadherin (VE-cad, Invitrogen, Thermo scientific, Rockford, IL, USA) to confirm their endothelial cell characteristics. For immunofluorescence, HUVECs were seeded at 8 × 10^4^ per well in 24-well plates and cultured for 24 h. The cell layers were washed with Hanks’ buffered salt solution (HBSS, HyClone, Beijing, China) at least three times and fixed with 4% (*w*/*v*) paraformaldehyde (Biosharp, Hefei, China) at 4 °C for 30 min. The cells were then permeabilized with 0.1% Triton X-100 (Life technologies, Frederick, MD, USA) and blocked with HBSS containing 1% (*w*/*v*) bovine serum albumin (BSA, Sigma-Aldrich, St. Louis, MO, USA) for 1 h at 37 °C before incubation with a primary Ab solution containing rabbit anti-vWF and anti-VE-cad (diluted in HBSS–0.5% (*w*/*v*) BSA, 1:100) for 2 h at 37 °C. To reveal the vWF and VE-cad, cells were washed with HBSS and then incubated with secondary Abs (Alexa 488 and Alexa 568 goat anti-rabbit IgG 1:1000, Molecular Probes, Life technologies, Eugene, OR, USA, respectively). To observe the location of VE-cad in HUVECs, cells were actin-stained with FITC-labeled phalloidin (Molecular Probes, Life technologies, Eugene, OR, USA). Finally, nuclei were revealed by incubating the cells with 1 μg mL^−1^ Hoechst dye 33342 (Thermo Fisher, Eugene, OR, USA) for 5 min at room temperature. Cells were then observed with a fluorescence microscope (DMI 3000B, Leica, Nussloch, Germany) and a laser scanning confocal microscope (LSCM, LSM700, Zeiss, Jena, Germany).

HUVECs (passages: 3–7) and HBMSCs (passages: 2–5) were used in this study. HUVECs and HBMSCs were either mono-seeded (40,000 cells/cm^2^) or co-seeded at a 4:1 ratio with direct contact. After 24 h culture, the cell culture media was changed. Mono-cultured HUVECs and HBMSCs were cultured in ECM and D-MEM, respectively, while the media ratio for co-culture was 1:1 ECM/D-MEM.

#### 2.2.2. Tube-Formation Assay

After plating Matrigel Matrix (Corning, Bedford, MA, USA) in 48-well plates and incubating at 37 °C to polymerize for 45 min, HUVECs alone and co-cultured HUVECs with HBMSCs were seeded for 30 min and then cultured with 10% extracts from PHA, FPHA0.25, FPHA0.50, and FPHA0.75, with 10% (*v*/*v*) serum-free α-MEM as a control. After incubation for 4 h, tube formation was observed by microscopy, and angiogenesis was assessed by calculating the following four parameters with online software (WimTube, Wimasis, Spain): total tubes, covered area, total tube length, and total loops. A tube is considered to be the part of a tubular structure found between two branching points or a branching point and a loose end, and total tubes refers to the number of tubes on the image. The percentage of the covered area is the percentage of tubular structures in the whole area of the image. It is calculated by dividing the total number of pixels of the image by the pixels that belong to the tubular structure. Total tube length was the length of the whole tubular structure in the pixels. A loop is an area of the background enclosed (or almost) by the tubular structure, while total loops means the number of loops on the image.

#### 2.2.3. Enzyme-Linked Immunosorbent Assay (ELISA) of VEGF

The secretion of VEGF-a released into the cell medium from HUVECs co-cultured with HBMSCs stimulated with 10% (*v*/*v*) extracts of PHA, FPHA0.25, and FPHA0.50 was determined using sandwich ELISA (RayBio kit ELH-VEGF-001, RayBiotech, Norcross, GA, USA) according to the protocol of the manufacturer.

Briefly, cells were cultured with different extracts in 24-well plates for 24 h. Supernatants from each group were collected (400 μL/well) and used immediately. Calibration standards (0~4000 pg/mL) and supernatants were pipetted into Ab-coated 96-wells. The VEGF present in the samples was detected through a biotinylated antihuman VEGF primary Ab bound to a second Ab that was linked to horseradish peroxidase (HRP) through a biotin–streptavidin bioconjugation. A tetramethylbenzidine (TMB) substrate solution was then added to the wells to determine the VEGF concentration on the basis of TMB-HRP reactions leading to the formation of a product exhibiting optical intensity, which was measured at 450 nm using a microplate reader (Tecan Infinite200, Tecan Group, Männedorf, Germany). A double logarithmic standard/calibration curve (*r*^2^ = 0.998) was obtained from curve fitting using 1-parameter logistic regression. A control group included blanks, i.e., Ab-coated wells solely inoculated with cell medium (not in contact with cells). The VEGF amounts determined from the blanks were subtracted from those obtained from the samples.

#### 2.2.4. *Vegf* Gene Expression

The effect of material extracts on the *vegf* gene expression of HUVECs co-cultured with HBMSCs stimulated with 10% (*v*/*v*) extracts of PHA, FPHA0.25, and FPHA0.50 for 24 h was studied using the quantitative real-time polymerase chain reaction assay (RT-qPCR). The total RNA of the cells was extracted using TriPure isolation reagent (Roche Diagnostics, Indianapolis, IN, USA). For the reverse transcription, complementary DNA was synthesized using a RevertAid First Strand cDNA Synthesis Kit (Thermo, OR, USA) following the manufacturer’s instructions. The primers used in the RT-qPCR assay were designed using software (v.3.0, Primer express, Applied Biosystems, Waltham, MA, USA) based on the reference sequence database provided by Genebank. FastStart Universal SYBR Green Master (Roche Diagnostics, Indianapolis, IN, USA) was used for the amplification and detection of cDNA targets on an ABI Prism 7500 Thermal Cycler (Applied Biosystems, Waltham, MA, USA). The mean cycle threshold (Ct) value of the target gene was normalized to the housekeeping gene glyceraldehyde 3-phosphate dehydrogenase (GAPDH). The results were shown as the fold change by comparing the mRNA expression levels of HUVECs alone and co-culture cells with the PHA and FPHA extracts using the ∆∆Ct method. The primer sequences were as follows: for *vegf*, Forward 5′-GAGGGCCTGGAGTGTGTGC-3′ and Reverse 5′-TCTTTCTTTGGTCTGCATTCAC-3′; and for *gapdh*, Forward 5′-GATTCCACCCATGGCAAATT-3′ and Reverse 5′-TCTCGCTCCTGGAAGATGGT-3′ (Table 1).

### 2.3. In Vivo Study

#### 2.3.1. Surgical Protocol

The study protocol was approved by the Committee on the Use of Live Animals for Teaching and Research, Sun Yat-sen University (IACUC-DB-15-1211) and maintained by a veterinarian at the Laboratory Animal Unit of the Faculty of Medicine, Sun Yat-sen University.

**Figure 1:**
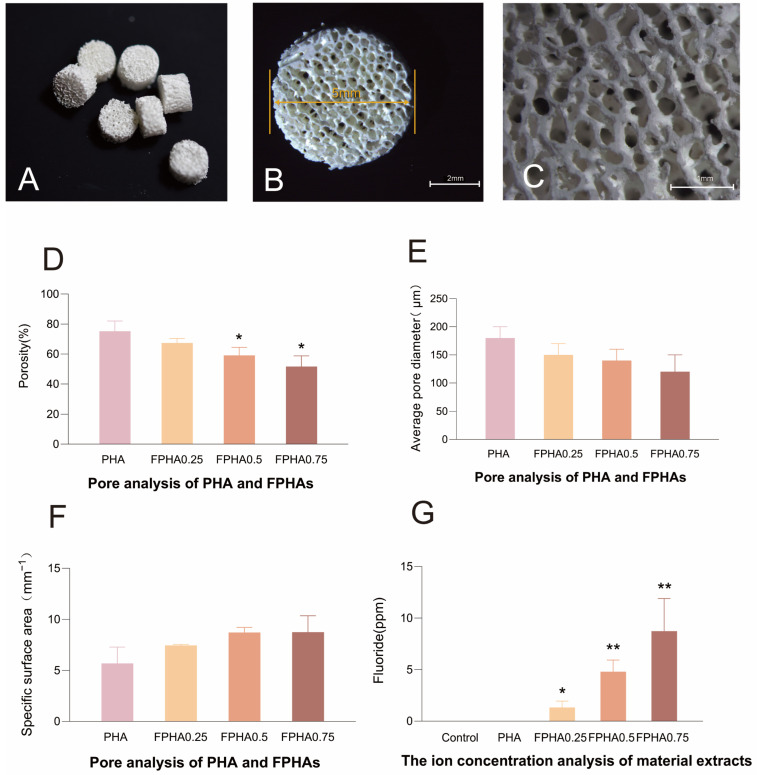
Preparation and physicochemical characterization of PHA and FPHA. (**A**) Preparation of bone blocks; (**B**,**C**) Morphological observation of bone blocks under a stereomicroscope; (**D**–**F**) Pore analysis of PHA and FPHA; (**G**) The ion concentration analysis of PHA and FPHA extracts. *: significant difference vs. control (*p* < 0.05). **: very significant difference vs. control (*p* < 0.01).

A total of 30 New Zealand rabbits (male, 5–6 months, 2.5–2.8 kg) were included in this study. For each rabbit, four defects of the same size (7 mm in diameter) were created with a trephine drill. Three of these defects were randomly divided and filled with equal amounts of PHA, FPHA0.25, and FPHA0.50, while the fourth one was left unfilled as a blank control. All surgical procedures were performed under general anesthesia. In detail, the animals were anesthetized with pentobarbital (30 mg/kg, I.V.) before the operation. The dorsal part of the scalp covering the calvaria was shaved and disinfected with iodophor gauze. After local anesthesia with 4% (*w*/*v*) articaine and adrenaline (1:100,000, Primacaine, Pierre Rolland, Bordeaux, France), a midline dermo-periosteal incision was made in the area of the nasoincisal suture. Subsequently, a full thickness flap was elevated to expose the calvarial bone. Four round defects were made in each calvarium with a trephine drill (external diameter 7 mm) under sufficient pre-cooled saline irrigation. The lateral wall of all defects and the dura mater underneath were carefully avoided to prevent damage, and the round defected bone chips were carefully removed; otherwise, the defect was excluded. The periosteum and skin were sutured with 4-0 nylon monofilament sutures (Figure 1).

#### 2.3.2. Postoperative Care and Sacrifice

Penicillin (30,000 U/kg, every 24 h for 3 days, I.M) was given after the operation to prevent infection. The health status, weight, and food consumption of the experimental animals were monitored and maintained until the time of sacrifice. After a healing period of 2, 6, and 16 weeks, the rabbits were euthanized with an overdose of barbiturate (150 mg/kg, I.V., Merck, Darmstadt, Germany).

#### 2.3.3. Histological Analysis

The calvaria containing the entire grafted area was retrieved in blocks, perfused with 4% (*w*/*v*) paraformaldehyde solution for immediate fixation, and was subsequently decalcified with 12.5% ethylenediaminetetraacetic acid (EDTA) for 8 weeks. Then, the blocks were dehydrated in ethanol, embedded in paraffin, and sectioned in the frontal plane through the middle of the defects. Three serial sections of 4 μm thickness were cut and then stained with hematoxylin and eosin (H&E) for histological analysis.

#### 2.3.4. Quantification of Blood Vessels

For the three samples from each group, the quantification of blood vessels was visualized and photographed with an upright microscope (DM 4000, Leica, Nussloch, Germany). New blood vessels were identified in H&E stained tissue section, by the defined lumens and the presence of red blood cells within their boundaries. Microvessel density (MVD) was calculated by the average number of microvessels in three random areas of an image at 100× magnification (ImageJ 1.50i, NIH, Bethesda, MD, USA) by a single-blinded researcher (Li, Z).

#### 2.3.5. Immunohistochemical Analysis

To stain for VEGF-a and PDGFR-β, tissue sections were treated with proteinase K (Sigma-Aldrich, St. Louis, MO, USA) for antigen recovery, washed with PBS, blocked with 5% BSA at room temperature for 30 min, and exposed to Abs for VEGF-a (5 μg/mL, Abcam, Cambridge, MA, USA) and *PDGFR-β* (20 μg/mL, Cambridge, MA, USA) overnight at 4 °C. Peroxidase activity was detected using the enzyme substrate 3-amino-9-ethyl carbazole. Negative controls were stained using the same protocol but without the primary Ab.

#### 2.3.6. Statistical Analysis

All data were presented as the mean ± standard deviation (SD). All error bars were based on the SDs of three samples used for each data point. A one-way analysis of variance with KW2 was used for multiple comparisons, while unpaired two-tailed Student’s *t*-tests were performed for comparisons between two groups. The level of significance was defined as *α* = 0.05. The calculations were performed with software (STATA, v. 14.0, College Station, TX, USA).

## 3. Results

The preliminary processing of the experimental raw porcine bone material included the removal of connective tissues, such as cartilage and periosteum, along with cortical bone, to preserve the natural cancellous bone structure. Chemical pre-treatment effectively eliminated most organic components derived from marrow lipids and blood proteins, yielding a material with a creamy yellow hue. After the first high-temperature sintering, the bone pieces took on a chalky white appearance and exhibited a porous interconnected architecture (Figure 1A–C). Micro-CT recorded and showed the porous spatial structure of the bone blocks and further analyzed it (Figure S1). The porosity of PHA and FPHA0.25 was approximately 70%, whereas FPHA0.50 and FPHA0.75 had slightly lower porosities of around 56% (Figure 1D). Specific surface area measurements ranged between 7 and 8 mm^−^^1^ (Figure 1F), with statistically significant inter-group differences in porosity; FPHA0.50 and FPHA0.75 displayed a notable decrease in porosity compared to PHA. The mean pore size for PHA was 180 ± 20 μm, while the pore sizes in FPHA0.25, FPHA0.50, and FPHA0.75 were reduced to 150 ± 20 μm, 140 ± 10 μm, and 120 ± 30 μm (Figure 1E), respectively, with a non-significant trend toward smaller pore sizes with increased fluorine doping. Specific surface area showed an increasing trend with fluorine concentration; however, the differences compared to PHA were not statistically significant. With an interconnectivity rate of 99.99%, these materials, derived from natural cancellous bone, exhibited minimal blind pores, indicating high porosity connectivity in both FPHA and PHA.

The ICP elemental analysis of PHA and FPHA extracts, α-MEM media as the control, the pH level, and fluorine ion concentration measurements showed pH levels consistently between 7 and 8. The fluorine ion concentrations in the FPHA0.25, FPHA0.50, and FPHA0.75 extracts were 1.34 ± 0.6 ppm, 4.81 ± 1.1 ppm, and 8.75 ± 3.2 ppm, respectively, increasing with higher doping levels. No fluorine ions were detected in fresh α-MEM or PHA extracts, as values fell below the detection range (10^−^^1^ to 10^−^^6^ ppm) (Figure 1G).

We successfully isolated and identified HUVECs and HBMSCs. As shown in Figures S2 and S3, these results confirm the isolated HUVECs’ endothelial phenotype and the HBMSCs’ multipotency. CCK-8 assays show that the FPHA0.75 extract displayed reduced proliferation compared to the control, suggesting that elevated fluorine in FPHA0.75 may inhibit HUVEC proliferation (Figure S4). Migration assays with the PDGFR inhibitor AC710 revealed a significant reduction in HUVEC migration across all groups, but FPHA0.25-treated HUVECs still showed increased migration compared to the control (Figure S5).

**Figure 2:**
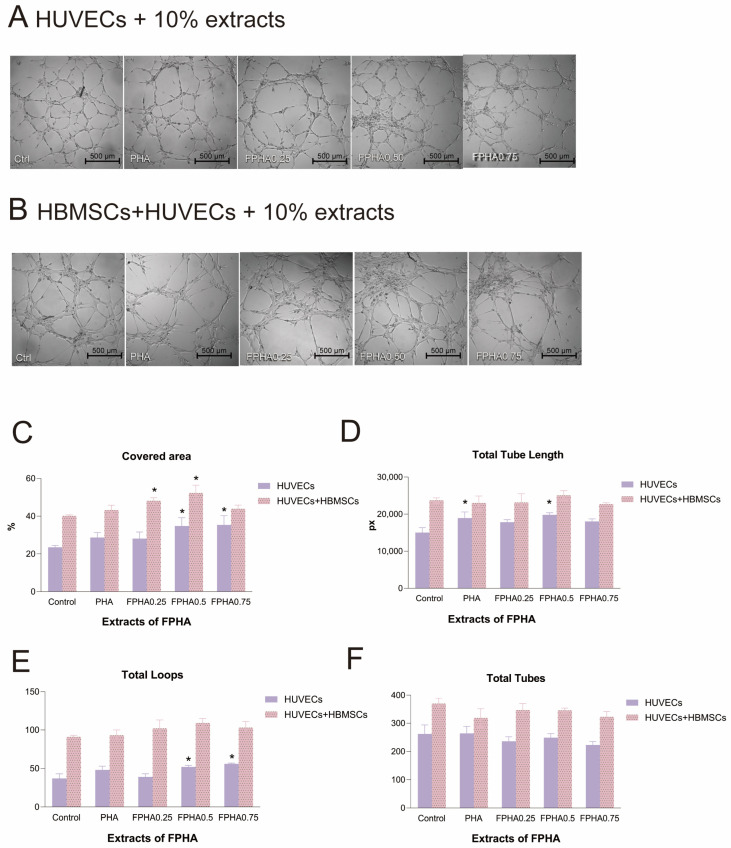
Optical images (at 50× magnification) of the in vitro angiogenesis assay of cells cultured on Matrigel in the presence of 10% PHA, FPHA0.25, FPHA0.50, and FPHA0.75 extracts for 4 h. (**A**) HUVECs alone; (**B**) HUVECs co-cultured with HBMSCs. Quantitative evaluation for tube formation after being cultured on Matrigel for 4 h in the presence of the extracts of PHA, FPHA0.25, FPHA0.50, and FPHA0.75: (**C**) Covered area; (**D**) Total tube length; (**E**) Total loops; (**F**) Total tubes; *: significant difference vs. control (*p* < 0.05).

Observing Matrigel assays with HUVECs and material extracts for 4 h demonstrated the formation of primary vascular structures, intermediate rings, and late-stage tubular networks (Figure 2A). A statistical analysis by Wimasis showed that the FPHA0.50 extract significantly enhanced the tubule-covered area, total tubule length, and loop count compared to the control (Figure 2C–E). However, the FPHA0.75 extract showed a downward trend in total loops and total tubes compared to control.

In Matrigel-based in vitro tubule formation assays, the co-culture of HUVECs and HBMSCs with material extracts yielded more extensive and complex tubular and network structures compared to HUVEC monocultures (Figure 2B), suggesting that there is a close interaction between HUVECs and HBMSCs, with the physiological activities of HBMSCs potentially influencing the proliferation and migration of HUVECs to some extent. Compared to the control group, FPHA0.25 and FPHA0.50 extract treatments significantly increased the tubular structure coverage area (Figure 2C). This further suggests that fluoride ions play a crucial role in regulating the interaction between HBMSCs and HUVECs. However, there were no significant differences in the tubule formation numbers between groups in both HUVEC monocultures and HUVEC-HBMSC co-cultures.

**Figure 3:**
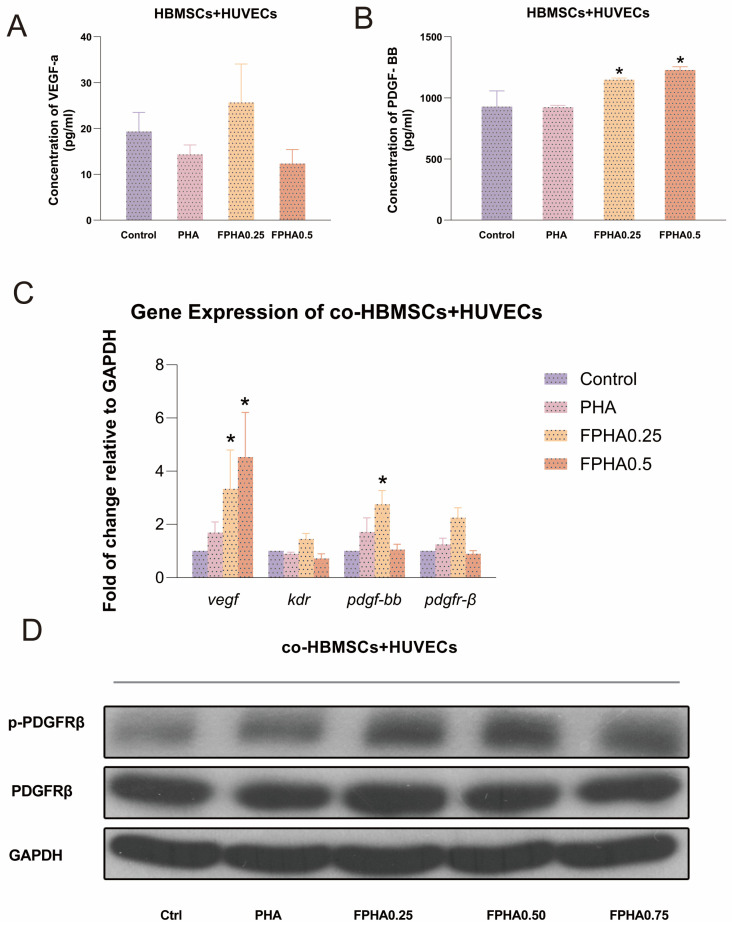
Influence of FPHA extract on the gene expression and secretory profile of HBMSCs and HUVECs in a co-culture model. (**A**) ELISA analysis of concentrations of VEGF-a in the supernatant of the co-cultured HBMSCs and HUVECs; (**B**) Concentrations of PDGF-BB in the supernatant of the co-cultured HBMSCs and HUVECs; (**C**) Expressions of the angiogenesis-related genes (*vegf* and its receptor kdr, *pdgf-bb* and its receptor *pdgfr-β* co-cultured with extracts of PHA, FPHA0.25, FPHA0.50, and FPHA0.75 for 24 h; (**D**) Western blot analysis of the co-cultured HBMSCs and HUVECs confirmed that both PDGFRβ and phosphorylation-PDGFRβ were present with or without extracts of FPHA (PHA, FPHA0.25, FPHA0.50, or FPHA0.75). *: significant difference vs. control (*p* < 0.05).

The effects of PHA, FPHA0.25, and FPHA0.50 extracts on the secretion of VEGF-a and PDGF-BB by HUVEC-HBMSC co-cultures for over 24 h are shown in Figure 3. The FPHA0.25 extract significantly increased VEGF-a secretion by HUVEC-HBMSC compared to the control. PDGF-BB concentrations after 24 h incubation with PHA, FPHA0.25, FPHA0.50, and the control were 928 ± 129, 923 ± 15, 1148 ± 14, and 1228 ± 26, respectively. The FPHA0.25 and FPHA0.50 extract groups showed significantly higher PDGF-BB secretion compared to the control (Figure 3B).

In the HUVEC-HBMSC co-culture system, FPHA0.25 and FPHA0.50 extracts significantly upregulated VEGF gene expression after 24 h (Figure 3C). Similarly, PDGF-BB gene expression was elevated by FPHA0.25 and FPHA0.75 treatments (Figure 3C).

Western blot analysis confirmed that FPHA extracts influenced PDGF-BB and PDGFR-β protein expression in HUVEC-HBMSC co-cultures. FPHA extracts upregulated PDGFR-β protein in HBMSCs while reducing its phosphorylation. In contrast, FPHA extracts enhanced PDGFR-β phosphorylation in HUVECs, with even more pronounced phosphorylation in the HUVEC-HBMSC co-culture system (Figure 3D).

**Figure 4:**
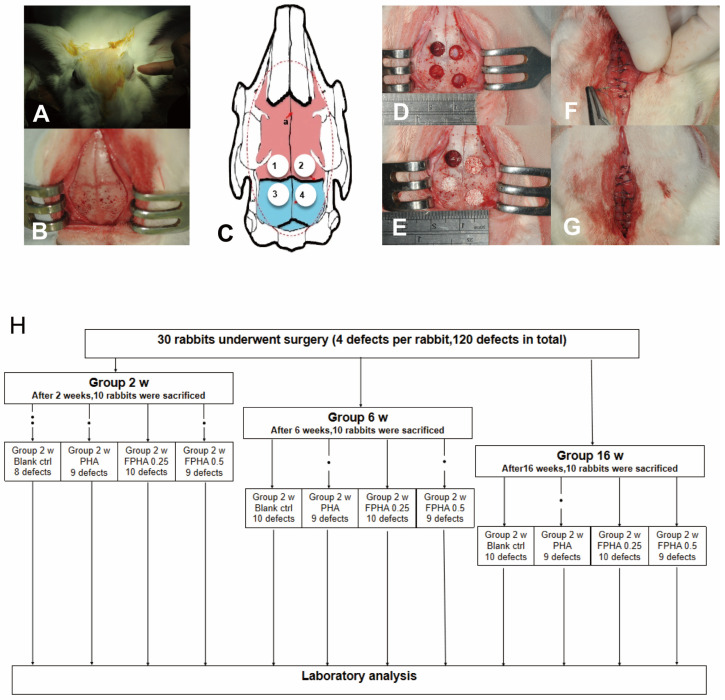
The surgical procedures: (**A**–**G**) The parietal bone was divided into four quadrants by the sagittal and coronal sutures (**C**) The schematic diagram of circular bone defect preparation divides the calvarial bone into four quadrants using the sagittal (a) and coronal sutures. The sites are labeled sequentially as ① (left anterior), ② (right anterior), ③ (left posterior), and ④ (right posterior). (**D**) in which a 7 mm diameter circular full-thickness defect was prepared; (**E**) defects were filled with bone graft material in accordance with the original anatomical shape of the skull; (**F**,**G**) covered and fixed implant material by the bilateral periosteum subcutaneous tissues and sutured the skin. Schematic diagram showing the animal and grafted defect groups. (**H**) Detailed grouping and experimental plan for the animal study.

In vivo analysis involved 30 New Zealand rabbits, yielding 120 cranial defect sites for testing bone substitute materials implantation. After excluding cases with dural injuries (7 rabbits, totaling 8 defects), 112 defect sites were retained across four groups: control, PHA, FPHA0.25, and FPHA0.50. Each group included samples for three observation periods: 2, 6, and 16 weeks, with a minimum of eight defects per period (Figure 4H). Postoperative animal health was stable, with normal feeding and excretion, and incisions showed primary healing without infection or dehiscence.

At 2 weeks post-surgery, defects in the control group exhibited only minor soft tissue coverage without bone formation. In the PHA and FPHA groups, fibrous connective tissue was observed on the material surface, with distinct borders between the defect margins and the material. At 6 and 16 weeks, materials appeared to be integrated with bone tissue, with indistinct boundaries on the cranial surface, although clearer boundaries were observed on the bottom of defect.

**Figure 5:**
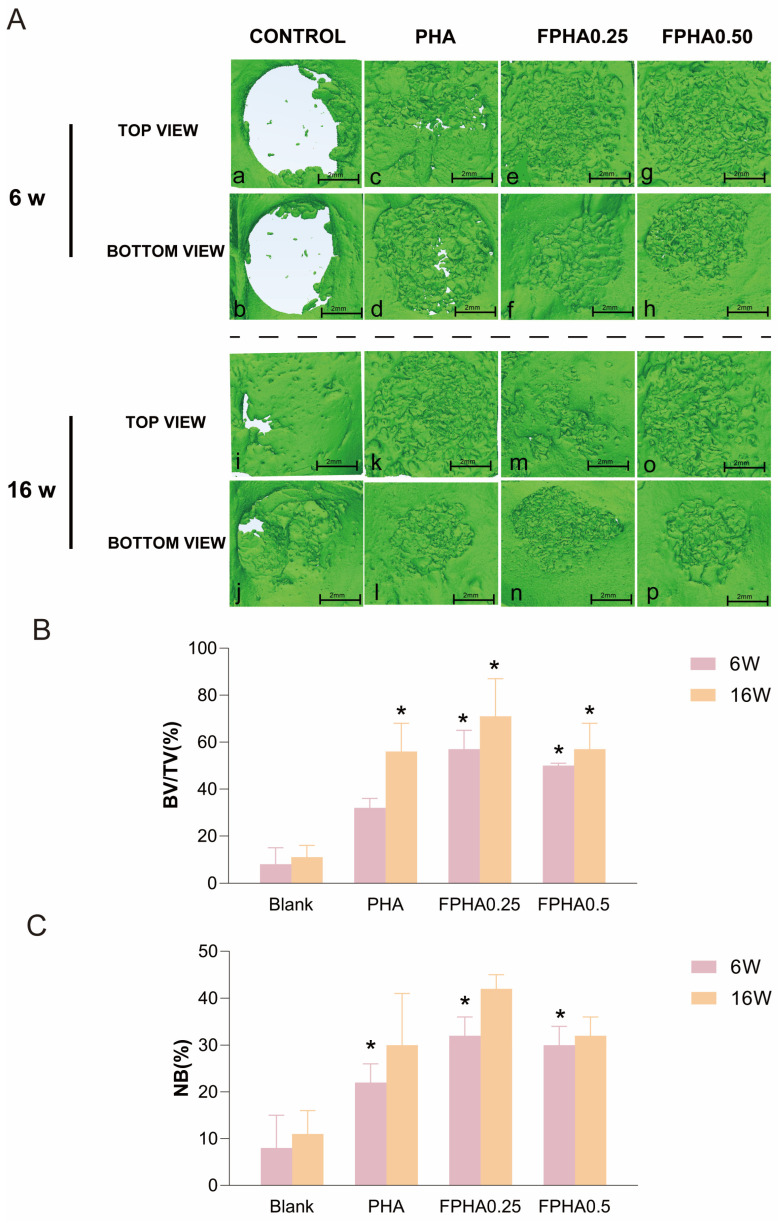
Micro-CT evaluation of bone formation in the cranial defect region of rabbits. (**A**) Three-dimensional reconstruction of rabbit calvarial bone defects ((**a**,**b**,**i**,**j**) were blank controls; (**c**,**d**,**k**,**l**) defects were grafted with PHA; (**e**,**f**,**m**,**n**) FPHA0.25; (**g**,**h**,**o**,**p**) FPHA0.50 granules; the upper two columns represented 6 w post-surgery and the lower two columns represented 16 w post-surgery). (**B**) Bone volume over total tissue volume (%) and (**C**). The percentage of new bone formation (%) within the bone defect of the blank control, PHA, FPHA0.25, and FPHA0.50 at 6 weeks and 16 weeks post-surgery. (* indicated *p* < 0.05).

Three-dimensional reconstructions of cranial defects at 6 weeks showed that control defects remained circular, with only minor bone growth at the edges (Figure 5A(a,b)). PHA-treated defects contained high-density graft material with evident porosity, displaying loose bone connectivity at the margin and center (Figure 5A(e,f)). FPHA0.25-treated defects exhibited smaller particles than PHA and showed enhanced new bone formation along the defect margins (Figure 5A(i,j)), indicating FPHA0.25’s capacity to induce early sidewall bone formation compared to PHA. FPHA0.50-treated defects showed similar outcomes, with more prominent early osteogenesis at the sidewalls (Figure 5A(m,n)). These findings suggest that ① early bone regeneration primarily occurs along the defect’s side and floor and that ② FPHA0.25 and FPHA0.50 show superior early osteogenic potential compared to the control and PHA groups.

At 16 weeks post-operation, three-dimensional reconstructed images of rabbit cranial defects were taken, as shown in Figure 5. In the blank control group (Figure 5A(c,d)), the margins of the bone wall appear more indistinct compared to those observed at 6 weeks, with newly formed bone progressing toward the defect center, although gaps in the bone wall indicated incomplete healing. In the PHA group (Figure 5A(g,h)), FPHA0.25 group (Figure 5A(k,l)), and FPHA0.50 group (Figure 5A(o,p)), defects were nearly filled with both material and new bone, with newly formed bone blurring the defect boundaries on the bottom, indicating defect reduction.

Further micro-CT quantitative analysis (Figure 5B,C) indicated that at 6 weeks, early defect healing showed BV/TV values of 57 ± 8% and 50 ± 1% for FPHA0.25 and FPHA0.50 groups, respectively, values significantly higher than those in the control group (8 ± 7%) and PHA group (32 ± 4%). At 6 weeks, new bone percentages in the PHA, FPHA0.25, and FPHA0.50 groups were 22 ± 4%, 32 ± 4%, and 30 ± 4%, respectively, values significantly greater than in the control group (8 ± 7%). At 16 weeks, the BV/TV% values for PHA, FPHA0.25, and FPHA0.50 were 56 ± 12%, 71 ± 16%, and 57 ± 11%, respectively, with all values being significantly higher than in the control group (11 ± 5%). New bone volume percentages at 16 weeks for these groups were 30 ± 11%, 42 ± 3%, and 32 ± 4%, with no significant differences found among them.

TRAP staining (Figure S6) at 2 weeks showed TRAP-positive macrophages surrounding the implant surfaces of PHA, FPHA0.25, and FPHA0.50, along with adjacent new bone and vascular structures. This observation suggests that in the initial stage of bone substitute material implantation, dynamic interactions between the material and the host tissue trigger angiogenic and osteogenic activities.

**Figure 6:**
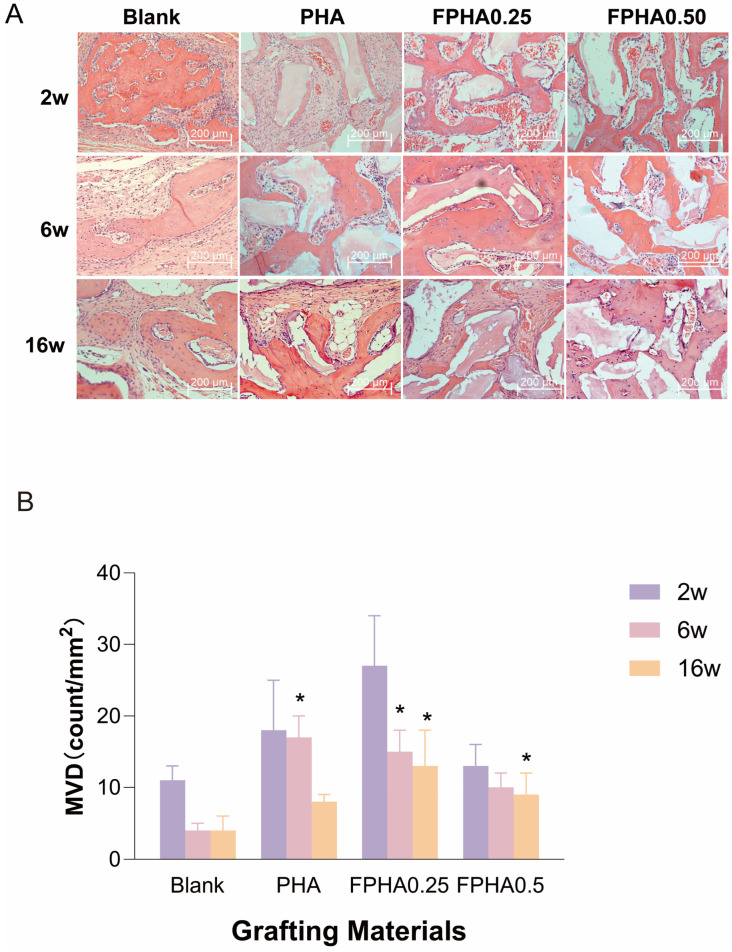
H&E staining for the decalcified sections of PHA, FPHA0.25, and FPHA0.50 implanted into calvarial defects of rabbits for 2, 6, and 16 weeks. (**A**) Photomicrographs at 200× magnification. (**B**) Microvessel density analysis after 2 w, 6 w, and 16 w post-implantation, * significant difference vs. blank control (*p* < 0.05).

Histological observations at 200× magnification (Figure 6A) showed homogeneous pink material filling defect areas, with decalcified white spaces and red-stained mineralized new bone containing shriveled osteocytes. Osteoblast linings were visible between mineralized bone and connective tissue, with marrow cavities occasionally seen around connective tissue. In all groups, no necrosis was observed, and materials were free of inflammatory encapsulation. Notably, FPHA0.25 and FPHA0.50 groups displayed more mineralized new bone than PHA, having close contact with or surrounding the material. At 2 weeks, abundant circularly arranged endothelial cells and blood cells inside vessels were visible in the newly formed vasculature; within the trabecular structures, osteoblasts were lined up along the material gaps, showing active proliferation and polymorphism. PHA exhibited similar bone formation patterns, but with relatively less mineralized new bone compared to FPHA0.25 and FPHA0.50. By 16 weeks, vascularization decreased markedly, while mineralized new bone increased. Mineralized bone was observed not only at defect edges but also within defect centers, with a thickening of bone trabeculae, showing no significant differences between material groups.

Histological analysis (Figure 6B) demonstrated higher microvascular density in defects repaired with PHA, FPHA0.25, and FPHA0.50 particles than in the blank control group. At 6 and 16 weeks, FPHA0.25 exhibited the highest microvascular density among all groups. At 16 weeks, FPHA0.50 also showed significantly enhanced microvascular formation compared to the control group.

Comparisons of microvascular density across different time points revealed significant differences between groups at 2 weeks post-surgery. However, the microvascular density exhibited a marked decreasing trend as healing progressed. The difference between 6 weeks and 2 weeks was not statistically significant, whereas a significant reduction in microvascular density was observed between 6 weeks and 16 weeks.

**Figure 7:**
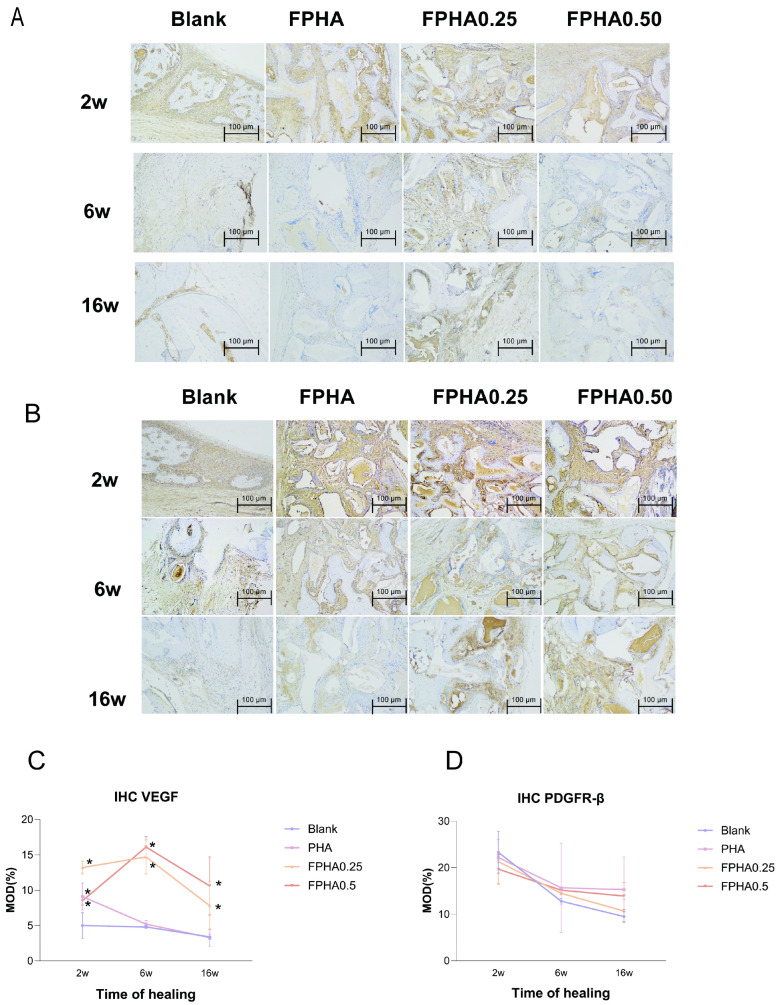
Representative photomicrographs of IHC staining. (**A**,**B**), respectively, show the VEGF and PDGFR-β distribution in the decalcified sections of the rabbit calvarial defects for 2, 6, and 16 weeks: the blank defect without grafting materials and defects grafted with PHA, FPHA0.25, FPHA0.50, and FPHA0.75. (**C**,**D**) Quantitative analysis for angiogenic factors VEGF and PDGFR-β present in the FPHA grafted calvaria defect of rabbits for 2, 6, and 16 weeks. * significant difference vs. blank control (*p* < 0.05).

Both VEGF and PDGFR-β showed positive expression across all groups, including the control, PHA, FPHA0.25, and FPHA0.50 groups (Figure 7A,B). In normal cortical and cancellous bone, VEGF positivity was primarily detected in osteoblasts, while osteocytes showed no VEGF expression. In the bone injury regions, VEGF staining was predominantly localized in cells surrounding newly formed bone trabeculae, with no VEGF expression observed in the mineralized matrix of the new bone.

Further quantitative analysis indicated an upward trend in VEGF expression from 2 to 6 weeks across both control and material groups, reaching a peak at 6 weeks before subsequently declining (Figure 7C). In contrast, PDGFR-β expression exhibited a gradual downward trend over time, with the overall positive expression rates across all groups and time points being consistently lower than those of VEGF (Figure 7D).

Among material groups, VEGF expression was significantly higher in the FPHA0.25 and FPHA0.50 groups than in the control group across all time points. In the PHA group, VEGF expression was significantly elevated compared to the control only at 2 weeks. Additionally, PDGFR-β expression in the FPHA0.25 group was significantly higher than in the control group in rabbit cranial defects.

## 4. Discussion

In the early stages following implant placement, host cells rely on the transport of nutrients and metabolites from surrounding host tissues to migrate onto the scaffold. This initial transport phase largely depends on diffusion, which originates from capillaries within the connective tissues adjacent to the implant. However, diffusion from these capillaries is effective only within a radius of 200 μm [23]. When scaffold materials exhibit low connectivity or small internal channels, or when blind pores are present, regions over 200 μm from the edge are inadequately reached, hindering oxygen, nutrient, and metabolite transport, which impedes bone regeneration. In this study, the macro-porosity and connectivity of PHA and FPHA scaffolds were characterized using the pore analysis module of the Mimics 16.0 software with Micro-CT imaging. Results indicate that fluorine ion doping preserves the 3D porous structure of biogenic hydroxyapatite, with PHA and FPHA0.25 exhibiting approximately 70% porosity, while FPHA0.50 and FPHA0.75 showed slightly lower porosity, around 56%. The pore rate and pore size showed a decreasing trend with increasing fluorine doping, with porosity ranging from 50 to 80% and pore sizes ranging from 120 to 150 μm. The specific surface area increased with the fluorine concentration, enhancing the surface area per unit volume of the material. Most hydroxyapatite ceramic bone substitutes used in previous studies report porosities of 60–80% and pore sizes of 100–800 μm [24,25], while the natural human cancellous bone has a pore size of 200–400 μm, and cortical bone is in the range of 1–100 μm [26]. In this study, PHA and FPHA scaffolds derived from porcine cancellous bone had a measured pore connectivity of 99.99%, essentially eliminating blind pores, with pore sizes between 120 and 180 μm. These pore sizes, resulting from fluorinated porcine cancellous bone-derived hydroxyapatite, are between those of human cancellous and cortical bone. Angiogenesis is closely related to the pore size in the scaffold and ultimately affects bone formation [27]. While the exact channel diameters within pores were not measured, 3D reconstructions from Micro-CT indicated internal channels with diameters approximating 100 μm, suitable for vascularization [28].

The activation, proliferation, and formation of tubular structures by HUVECs represent the initial phase of angiogenesis. In this study, we evaluated the effects of PHA and FPHA extracts on the proliferative activity of HUVECs.

At the 1 day observation point, all extract groups showed no inhibitory effect on HUVEC proliferation, except for the 10% PHA extract, which exhibited a slight inhibitory effect. However, at day 5 and day 7, the gradual release of fluoride ions from FPHA incorporated in the extracts led to an increase in the fluoride concentration in the medium. Notably, the release of fluoride ions from the 10% FPHA0.75 extract exerted a cytotoxic effect on HUVECs, impairing their proliferation.

The tube formation of HUVECs is considered essential in angiogenesis because it represents the organization of endothelial cells into a network that simulates capillaries [29]. In the tube formation assay, both FPHA0.25 and FPHA0.50 extracts enhanced the formation of tubular structures, whether in HUVEC monocultures or HUVEC-HBMSC co-cultures. However, the FPHA0.75 extract reduced the efficiency of tube formation, inhibiting early angiogenic activity.

These findings suggest that the regulatory effects of fluoride ions on HUVECs are dose-dependent. At lower concentrations, fluoride ions promote HUVEC proliferation and tube formation, whereas higher concentrations inhibit cell growth and proliferation. This observation aligns with the previous literature [30,31]. Given the potential cytotoxicity of the FPHA0.75 extract observed in this study, this group was excluded from subsequent ELISA and PCR assays and in vivo experiments.

VEGF is a crucial growth factor in regulating vascular development and angiogenesis [32]. Studies have shown a correlation between postmenopausal osteoporosis and reduced VEGF levels [33]. Mice with reduced VEGF levels in osteoblasts exhibit osteoporotic phenotypes postnatally [34]. Bone, as a highly vascularized tissue, depends on vascularization for bone formation and remodeling. VEGF plays a key role in bone development and postnatal bone defect repair, supporting the functional proximity and dependency between vascular and osteogenic cells [35]. In this study, although VEGF mRNA was upregulated in HUVEC-HBMSC co-cultures stimulated with extract, only a small amount of VEGF was found in the supernatant of HUVEC-HBMSC co-cultures, suggesting that detectable VEGF-a in the culture media is likely derived from mesenchymal cells rather than endothelial cells [14,36]. This observation aligns with other studies indicating that bone marrow mesenchymal and osteoprogenitor cells are the primary sources of VEGF in the bone environment, despite certain studies showing contradictory findings [37].

Beyond endothelial cells, perivascular and stromal cells play vital roles in angiogenesis. PDGF-BB and its receptor PDGFR-β are crucial for stabilizing vascular structures and supporting angiogenesis [38]. PDGFR-β-positive bone marrow-derived mesenchymal cells can differentiate into perivascular cells, enhancing vascular stability and survival. PDGF expression promotes vascular wall cell coverage, supporting angiogenesis, whereas the inhibition of PDGFR signaling reduces perivascular cell recruitment, leading to vessel dilation and increased endothelial apoptosis [39]. In the HUVEC-HBMSC co-culture system, 24 h exposure to FPHA0.50 extracts significantly increased *pdgf-bb* gene expression levels. Western blot analysis confirmed FPHA’s effect on PDGF-BB and PDGFR-β protein expression, showing that FPHA extracts upregulated PDGFR-β protein expression in HBMSCs, though the phosphorylation levels were reduced, suggesting a weakened downstream signaling cascade. In contrast, FPHA0.25 and FPHA0.50 extracts in HUVEC-HBMSC co-cultures notably enhanced PDGFR-β phosphorylation. Various factors influence receptor–ligand affinity, with the tyrosine phosphorylation of receptors promoting ligand binding, while dephosphorylation can impair binding affinity.

FPHA promotes cellular function by stimulating HBMSCs to secrete VEGF, regulating the VEGF-KDR pathway and modulating endothelial cell function through PDGFR-β phosphorylation. FPHA also stimulates HUVECs to secrete PDGF-BB, which acts on HBMSCs via PDGFR-β phosphorylation to facilitate endothelial cell migration and maturation. In other biological studies, NaF has been identified as an activator initiating stimulus–response cascades, including MAP kinase (mitogen-activated protein, MAP), Erk-1, and Erk-2 [40,41]. The fluoride regulation of cellular activity is closely associated with MAP kinase signaling pathways. Whether the FPHA stimulation of the VEGF/KDR downstream pathway activates MAP kinase cascades through Ras/MEKK/MEK-induced Erk activation requires further investigation. The interaction between PDGF-BB and PDGFR-β induces PDGFR-β phosphorylation and activates the PI3K pathway. The PI3K/Akt pathway is a critical regulatory mechanism involved in cellular proliferation, migration, differentiation, and angiogenesis. You et al. proposed that Foxc2 promotes angiogenesis in BMSCs through PI3K and ERK pathways [42]. Osteoblast migration and proliferation have been identified as fundamental steps in bone healing. Previous studies confirmed that G-protein-coupled receptor kinase interacting protein 1 (GIT1) is a key regulator of bone mass and osteoblast migration, although its upstream signaling pathways and downstream effectors remain unclear [43]. It has also been found that PDGF promotes GIT1 tyrosine phosphorylation in osteoblasts, and that focal adhesion kinase (FAK) expression associated with GIT1 increases in the localized adhesion of osteoblasts [44]. In the bone regeneration microenvironment, FPHA triggers specific intracellular signaling cascades, activating endothelial cell migration-related proteins and inducing angiogenesis to regulate bone regeneration. This study proposes that FPHA regulates endothelial cell function through the VEGF-KDR signaling pathway and PDGFR-β phosphorylation, providing a new perspective for exploring the mechanisms of FPHA in osteogenesis and angiogenesis.

Histological observations in this study reveal evidence of both osteoinduction and osteoconduction. Micro-CT quantitative analysis showed that at 6 weeks post-operation, BV/TV in the FPHA0.25 and FPHA0.50 groups was significantly higher than in the PHA and control groups, suggesting enhanced bone regeneration through osteoconductive guidance by PHA and FPHA materials. The early-stage bone repair process with fluorinated hydroxyapatite (FPHA0.25 and FPHA0.50) was particularly effective at promoting new bone formation. At 16 weeks, new bone volumes did not significantly differ among materials, suggesting that PHA and FPHA materials yield comparable bone formation support in later stages of bone repair. Micro-CT 3D reconstructions revealed that, in the early healing stage, the newly formed tissue surrounding the defect margins was more abundant than at the defect center, likely due to nutrient and blood supply limitations within the scaffold’s center. Bone induction occurs with a spatial gradient, as larger bone defects experience reduced blood supply and nutrient diffusion beyond the 200 μm limit from scaffold peripheries. Additionally, chemotactic factors released from injured sites attract mesenchymal stem cells from periosteal and perivascular sources, creating a growth factor gradient that stimulates early-stage vascularization and bone formation. Effective bone regeneration was observed with low-fluoride concentration FPHA over a 16-week period. However, extending the observation period would be more beneficial. Although significant differences in BV/TV and histological sections were observed among groups at 2, 6, and 16 weeks, the stability of the material’s osteogenic and angiogenic effects requires further investigation. Moreover, as new bone formation is a continuous process, extending the experimental period would enable the further observation of new bone formation patterns through Micro-CT reconstruction, the calculation of BV/TV, and the assessment of indicators such as trabecular morphology density and neovascularization in histological sections. In this experiment, a 16-week observation period was selected based on the following professional considerations: the bone regeneration process in rabbits is relatively prolonged; and for extensive bone defects such as cranial defects, it typically requires 12–16 weeks to comprehensively evaluate new bone formation, material degradation, and bone tissue integration, thereby fully capturing the early biological responses induced by the material [45]. As a non-load-bearing bone, rabbit cranial bone exhibits limited healing capacity. In a critical-sized defect (CSD) model, a 16-week period effectively evaluates the rate of new bone formation, defect filling, and the biocompatibility and degradation properties of the material [46]. The bone healing process includes the inflammatory, repair, and remodeling phases, with critical milestones for the repair and remodeling phases occurring after 12 weeks. A 16-week observation period encompasses the primary stages of bone repair and enables the comprehensive evaluation of mid- to long-term outcomes. Furthermore, as the rate of bone regeneration in rabbit cranial bone is comparable to that in human cranial bone, a 16-week animal study period holds significant potential for clinical translation [40].

Immunohistochemical staining for VEGF in histological sections indicated that undifferentiated mesenchymal cells and differentiated osteoblasts around FPHA implants responded to VEGF antibodies, suggesting that VEGF expression supports an environment conducive to vascularization and bone regeneration. This finding aligns with previous research [47] indicating that osteogenic-related cells involved in bone healing, such as osteoprogenitor and mature osteoblasts [48,49], express VEGF. VEGF-positive staining was prominent in areas rich in newly formed vasculature within connective tissue and marrow cavities. Notably, VEGF staining was localized to cells surrounding new bone trabeculae, with no positive expression detected within the mineralized bone matrix. Quantitative analysis revealed a correlation between VEGF expression and fluorine concentration in FPHA, with FPHA0.25 and FPHA0.50 promoting VEGF expression. Studies on VEGF knockout models show osteoporotic changes in the bones of knockout mice, while VEGF-silenced mesenchymal cells tend toward adipogenic phenotypes [33]. Additionally, BMSCs exhibit reduced osteogenic differentiation upon VEGFR (KDR) inhibition, highlighting the roles of RUNX2 and PPARγ2 in VEGF-mediated osteogenic regulation. Since endogenous VEGF plays a critical role in bone regeneration, with VEGF knockout mice exhibiting high mortality and non-compensable bone defects even with exogenous VEGF supplementation [50], these results suggest that scaffold modifications that stimulate endogenous VEGF expression are more advantageous for vascularization than exogenous VEGF supplementation.

Histological analysis in this study shows that in the early phase of bone tissue injury (2 weeks), an active inflammatory response rapidly occupies the injury site, with an influx of blood cells, platelets, macrophages, and other inflammatory cascade-associated cells. This inflammatory response effectively isolates the injured area as an avascular region, thus preventing interference with or the infection of other body regions. Within this localized “isolated” environment, platelets and macrophages release numerous bioactive molecules, including PDGF-BB, that are crucial for bone repair [51]. Immunohistochemical staining for PDGFR-β in this study revealed concentrated PDGFR-β expression surrounding FPHA implants and vasculature. PDGFR-β-positive BMSCs, induced by PDGF-BB, migrated to perivascular areas and infiltrated the vascular outer membrane, differentiating into PDGFR-β-positive pericytes of mesenchymal origin around endothelial cells. These pericytes extended and stabilized newly formed vessels, supporting the maturation of neovasculature within the bone defect microenvironment, which in turn promotes bone regeneration. Moreover, pericytes can differentiate into osteoprogenitor cells, enhancing osteogenesis. Osteogenic cells in bone defects mainly derive from vascular-associated osteoprogenitors (osteoblast precursors) rather than mature osteoblasts from the periosteum. Thus, even if periosteum covers the defect center, osteogenesis in this area predominantly relies on the infiltration of PDGFR-β-positive cells along the defect margins through vessel extension, a concept partially supported by the faster osteogenesis observed in peripheral regions over the defect center in this study [34].

Additionally, the differences in PDGFR-β expression correlated with the level of fluorine doping in FPHA, as FPHA0.25 and FPHA0.50 demonstrated elevated levels of this angiogenesis-associated protein, providing evidence for optimal fluorine-doped FPHA in promoting in vivo vascularization, as presented in the previous chapter. PDGF-BB participates in multiple processes within this framework, not only stimulating local vascular formation but also upregulating osteogenic molecule levels, guiding their spatial distribution to accelerate bone formation. FPHA supports this coupling effect by stimulating endothelial cells to secrete PDGF-BB, thereby enhancing PDGFR-β expression in both endothelial and mesenchymal cells. This modulation of osteogenic-related cytokine distribution in the bone regeneration microenvironment facilitates coordinated vascularization and osteogenesis.

The 16-week observation period may be insufficient to comprehensively assess the long-term stability of vascular structures and bone integration. Although the rabbit cranial defect model offered valuable insights, it cannot fully replicate the complex biomechanical and systemic conditions present in human bone regeneration. The study concentrated on specific fluorine concentrations and their effects on VEGF and PDGF pathways, leaving additional molecular mechanisms insufficiently investigated. The absence of biomechanical testing constrains the applicability of these findings to load-bearing bones. Future studies are prepared to extend the observation period to 24–52 weeks to evaluate long-term bone regeneration and employ advanced animal models, such as large animals, to better simulate human conditions. Advanced methodologies, such as high-throughput sequencing and biomechanical testing, should be employed to investigate additional signaling pathways and assess the mechanical properties of regenerated bone. Optimizing fluorine concentrations is critical to achieving a balance between promoting osteogenesis and minimizing cytotoxicity.

## 5. Conclusions

The fluorinated porcine bone-derived hydroxyapatite (FPHA) prepared in this study retained the natural porous structure of biological cancellous bone and released F^−^ ions when immersed in cell culture medium. The extraction solutions of FPHA0.25 and FPHA0.50 promoted the formation of capillary-like tubes by human umbilical vein endothelial cells (HUVECs), with FPHA0.25 significantly upregulating *vegf* mRNA and VEGF protein levels in co-cultured human bone marrow mesenchymal stem cells (HBMSCs). Additionally, FPHA0.25 and FPHA0.50 upregulated *pdgf-bb* mRNA and PDGF-BB protein levels in HUVECs. In vivo experiments using a rabbit cranial defect model demonstrated that FPHA0.25 promoted early bone formation and angiogenesis in the defect area, enhanced VEGF secretion, and increased PDGFR-β expression in endothelial and mesenchymal cells. These findings suggest that FPHA with an optimal fluorine concentration (FPHA0.25) is a bone substitute material with significant angiogenic potential.

## Figures and Tables

**Table 1:** Primers used for rt-qPCR.

Target Gene	Forward Primer Sequence (5′-3′)	Reverse Primer Sequence (5′-3′)
*pdgf-bb*	CTCTCTGCTGCTACCTGCGTCT	CATCAAAGGAGCGGATCGAG
*pdgfr-β*	GTGGTTGAGAGCGGCTACGT	AGGCCTCGAACACTACCTGC
*vegf*	GAGGGCCTGGAGTGTGTGC	TCTTTCTTTGGTCTGCATTCAC
*kdr*	AGCGGCAAATGTGTCAGCTT	AATTTCAGGACCCCTGGTCAC
*GAPDH*	GATTCCACCCATGGCAAATT	TCTCGCTCCTGGAAGATGGT

## Data Availability

The data presented in this study are available upon reasonable request from the corresponding author.

## Supplementary Materials

The following supporting information can be downloaded at: https://www.mdpi.com/article/10.3390/bioengineering11121287/s1, Figure S1. Porosity analysis on the three dimension reconstruction of representitive Micro-CT data of FPHA0.25 block; Figure S2. HUVECs were characterized by contrast microscope observation; Figure S3. Multipotent differentiation potency and surface marker characterization and confirmation of HBMSCs; Figure S4. CCK-8 assay on the proliferation of HUVECs on days 1, 3, 5 and 7 cultured with 10% PHA and FPHA extracts; Figure S5. Representitive micrographs on the migration of HUVECs chemotacted by PHA/FPHA extracts at different concentration; Figure S6. TRAP+ osteoclast resided surrounding the residual materials and microvessels 2 weeks postsugery.

## References

[1] Zhou H., Lee J. (2011). Nanoscale Hydroxyapatite Particles for Bone Tissue Engineering. Acta Biomater..

[2] Ielo I., Calabrese G., Luca G., Conoci S. (2022). Recent Advances in Hydroxyapatite-Based Biocomposites for Bone Tissue Regeneration in Orthopedics. Int. J. Mol. Sci..

[3] Zaffarin A., Ng S., Ng M., Hassan H., Alias E. (2021). Nano-Hydroxyapatite as a Delivery System for Promoting Bone Regeneration In Vivo: A Systematic Review. Nanomaterials.

[4] Zhang H., Huang H., Hao G., Zhang Y., Ding H., Fan Z., Sun L. (2021). 3D Printing Hydrogel Scaffolds with Nanohydroxyapatite Gradient to Effectively Repair Osteochondral Defects in Rats. Adv. Funct. Mater..

[5] Xing J., Peng X., Li A., Chen M., Ding Y., Xu X., Yu P., Xie J., Li J. (2021). Gellan Gum/Alginate-Based Ca-Enriched Acellular Bilayer Hydrogel with Robust Interface Bonding for Effective Osteochondral Repair. Carbohydr. Polym..

[6] Zhang Y., Cui Y., Tian J., Chen X., Xu T., Liu J., Xu Y. (2022). Nanohydroxyapatite Hydrogel Can Promote the Proliferation and Migration of Chondrocytes and Better Repair Talar Articular Cartilage. Comput. Math. Methods Med..

[7] Aghyarian S., Rodriguez L.C., Chari J., Bentley E., Kosmopoulos V., Lieberman I.H., Rodrigues D.C. (2014). Characterization of a New Composite PMMA-HA/Brushite Bone Cement for Spinal Augmentation. J. Biomater. Appl..

[8] Karpiński R., Szabelski J., Krakowski P., Jonak J., Falkowicz K., Jojczuk M., Nogalski A., Przekora A. (2024). Effect of Various Admixtures on Selected Mechanical Properties of Medium Viscosity Bone Cements: Part 2—Hydroxyapatite. Compos. Struct..

[9] Cancedda R., Giannoni P., Mastrogiacomo M. (2007). A tissue engineering approach to bone repair in large animal models and in clinical practice. Biomaterials.

[10] Niu Y., Chen L., Wu T. (2023). Recent advances in bioengineering bone revascularization based on composite materials comprising hydroxyapatite. Int. J. Mol. Sci..

[11] Ma J., Wu S., Liu J., Liu C., Ni S., Dai T., Wu X., Zhang Z., Qu J., Zhao H. (2022). Synergistic effects of nanoattapulgite and hydroxyapatite on vascularization and bone formation in a rabbit tibia bone defect model. Biomater. Sci..

[12] Lv N., Zhou Z., Hou M., Hong L., Li H., Qian Z., Gao X., Liu M. (2024). Research progress of vascularization strategies of tissue-engineered bone. Front. Bioeng. Biotechnol..

[13] Kang Y., Kim S., Fahrenholtz M., Khademhosseini A., Yang Y. (2013). Osteogenic and angiogenic potentials of monocultured and co-cultured human-bone-marrow-derived mesenchymal stem cells and human-umbilical-vein endothelial cells on three-dimensional porous beta-tricalcium phosphate scaffold. Acta Biomater..

[14] Pedersen T.O., Blois A.L., Xue Y., Xing Z., Sun Y., Finne-Wistrand A., Lorens J.B., Fristad I., Leknes K.N., Mustafa K. (2014). Mesenchymal stem cells induce endothelial cell quiescence and promote capillary formation. Stem Cell Res. Ther..

[15] Bose S., Tarafder S. (2012). Calcium phosphate ceramic systems in growth factor and drug delivery for bone tissue engineering: A review. Acta Biomater..

[16] Shields L.B., Raque G.H., Glassman S.D., Campbell M., Vitaz T., Harpring J., Shields C.B. (2006). Adverse effects associated with high-dose recombinant human bone morphogenetic protein-2 use in anterior cervical spine fusion. Spine.

[17] LeGeros R.Z., Parsons J.R., Daculsi G., Driessens F., Lee D., Liu S.T., Metsger S., Peterson D., Walker M. (1988). Significance of the porosity and physical chemistry of calcium phosphate ceramics. Ann. N. Y. Acad. Sci..

[18] Liu G., Zhang W., Jiang P., Li X., Liu C., Chai C. (2012). Role of nitric oxide and vascular endothelial growth factor in fluoride-induced goitrogenesis in rats. Environ. Toxicol. Pharmacol..

[19] Birgani Z.T., Gharraee N., Malhotra A., van Blitterswijk C.A., Habibovic P. (2016). Combinatorial incorporation of fluoride and cobalt ions into calcium phosphates to stimulate osteogenesis and angiogenesis. Biomed. Mater..

[20] Liu Q., Chen Z., Gu H., Chen Z. (2012). Preparation and characterization of fluorinated porcine hydroxyapatite. Dent. Mater. J..

[21] Li H., Chang J. (2013). Stimulation of proangiogenesis by calcium silicate bioactive ceramic. Acta Biomater..

[22] (2018). Biological Evaluation of Medical Devices—Part 1: Evaluation and Testing Within a Risk Management Process.

[23] Lu Y., Hu D., Ying W. (2021). A fast numerical method for oxygen supply in tissue with complex blood vessel network. PLoS ONE.

[24] Karageorgiou V., Kaplan D. (2005). Porosity of 3D biomaterial scaffolds and osteogenesis. Biomaterials.

[25] Tzavellas A.N., Katrilaka C., Karipidou N., Kanari M., Pitou M., Koliakos G., Cheva A., Choli-Papadopoulou T., Aggeli A., Tsiridis E. (2024). The “Forgotten” Hydroxyapatite Crystals in Regenerative Bone Tissue Engineering: A Critical Review. Crystals.

[26] LeGeros R.Z. (2002). Properties of osteoconductive biomaterials: Calcium phosphates. Clin. Orthop. Relat. Res..

[27] Freitas P., Kishida R., Hayashi K., Tsuchiya A., Shimabukuro M., Ishikawa K. (2022). Fabrication and histological evaluation of porous carbonate apatite blocks using disodium hydrogen phosphate crystals as a porogen. J. Biomed. Mater. Res. A.

[28] Bobbert F., Zadpoor A.A. (2017). Effects of bone substitute architecture and surface properties on cell response, angiogenesis, and structure of new bone. J. Mater. Chem. B.

[29] Varinská L., Fáber L., Kello M., Petrovová E., Balážová Ľ., Solár P., Čoma M., Urdzík P., Mojžiš J., Švajdlenka E. (2018). β-Escin effectively modulates HUVECs proliferation and tube formation. Molecules.

[30] Szczepański M., Kamianowska M., Kamianowski G. (2012). Effects of fluorides on apoptosis and activation of human umbilical vein endothelial cells. Oral Dis..

[31] Li J., Jin L., Zhang H. (2007). Effect of fluoride on proliferation of human blood vessel endothelial cells in culture. J. Environ. Health.

[32] Florek K., Mendyka D., Gomułka K. (2024). Vascular endothelial growth factor (VEGF) and its role in the cardiovascular system. Biomedicines.

[33] Senel K., Baykal T., Seferoglu B., Altas E.U., Baygutalp F., Ugur M., Kiziltunc A. (2013). Circulating vascular endothelial growth factor concentrations in patients with postmenopausal osteoporosis. Arch. Med. Sci..

[34] Liu Y., Berendsen A.D., Jia S., Lotinun S., Baron R., Ferrara N., Olsen B.R. (2012). Intracellular VEGF regulates the balance between osteoblast and adipocyte differentiation. J. Clin. Investig..

[35] Maes C., Kobayashi T., Selig M.K., Torrekens S., Roth S.I., Mackem S., Carmeliet G., Kronenberg H.M. (2010). Osteoblast precursors, but not mature osteoblasts, move into developing and fractured bones along with invading blood vessels. Dev. Cell.

[36] Hu K., Olsen B.R. (2016). Osteoblast-derived VEGF regulates osteoblast differentiation and bone formation during bone repair. J. Clin. Investig..

[37] Li H., Daculsi R., Grellier M., Bareille R., Bourget C., Remy M., Amedee J. (2011). The role of vascular actors in two-dimensional dialogue of human bone marrow stromal cell and endothelial cell for inducing self-assembled networks. PLoS ONE.

[38] Ruan J., Luo M., Wang C., Fan L., Yang S.N., Cardenas M., Geng H., Leonard J.P., Melnick A., Cerchietti L. (2013). Imatinib disrupts lymphoma angiogenesis by targeting vascular pericytes. Blood.

[39] Carmeliet P. (2005). Angiogenesis in life, disease and medicine. Nature.

[40] Matsubara H., Hogan D.E., Morgan E.F., Mortlock D.P., Einhorn T.A., Gerstenfeld L.C. (2012). Vascular Tissues Are a Primary Source of BMP2 Expression During Bone Formation Induced by Distraction Osteogenesis. Bone.

[41] Paul A., Wilson S., Belham C.M., Robinson C.J., Scott P.H., Gould G.W., Plevin R. (1997). Stress-Activated Protein Kinases: Activation, Regulation and Function. Cell. Signal..

[42] You W., Gao H., Fan L., Duan D., Wang C., Wang K. (2013). Foxc2 Regulates Osteogenesis and Angiogenesis of Bone Marrow Mesenchymal Stem Cells. BMC Musculoskelet. Disord..

[43] Michaelis U.R. (2014). Mechanisms of Endothelial Cell Migration. Cell. Mol. Life Sci..

[44] Ren Y., Yu L., Fan J., Rui Z., Hua Z., Zhang Z., Zhang N., Yin G. (2012). Phosphorylation of GIT1 Tyrosine 321 Is Required for Association with FAK at Focal Adhesions and for PDGF-Activated Migration of Osteoblasts. Mol. Cell. Biochem..

[45] Wang J., Wang L., Zhou Z., Lai H., Xu P., Liao L., Wei J. (2016). Biodegradable Polymer Membranes Applied in Guided Bone/Tissue Regeneration: A Review. Polymers.

[46] Oryan A., Alidadi S., Moshiri A., Maffulli N. (2014). Bone Regenerative Medicine: Classic Options, Novel Strategies, and Future Directions. J. Orthop. Surg. Res..

[47] Street J., Bao M., DeGuzman L., Bunting S., Peale F.V., Ferrara N., Steinmetz H., Hoeffel J., Cleland J.L., Daugherty A. (2002). Vascular endothelial growth factor stimulates bone repair by promoting angiogenesis and bone turnover. Proc. Natl. Acad. Sci. USA.

[48] Riddle R.C., Khatri R., Schipani E., Clemens T.L. (2009). Role of Hypoxia-Inducible Factor-1α in Angiogenic-Osteogenic Coupling. J. Mol. Med..

[49] Kakudo N., Kusumoto K., Wang Y.B., Iguchi Y., Ogawa Y. (2006). Immunolocalization of Vascular Endothelial Growth Factor on Intramuscular Ectopic Osteoinduction by Bone Morphogenetic Protein-2. Life Sci..

[50] Lee S., Chen T.T., Barber C.L., Jordan M.C., Murdock J., Desai S., Ferrara N., Nagy A., Roos K.P., Iruela-Arispe M.L. (2007). Autocrine VEGF Signaling Is Required for Vascular Homeostasis. Cell.

[51] Chen Y., Jiang L., Lyu K., Lu J., Long L., Wang X., Liu T., Li S. (2022). A Promising Candidate in Tendon Healing Events—PDGF-BB. Biomolecules.

