# Supplementary material for: Fluorinated Porcine Bone-Derived Hydroxyapatite Promotes Vascularized Osteogenesis by Coordinating Human Bone Marrow Mesenchymal Stem Cell/Human Umbilical Vein Endothelial Cell Complexes
Source: Bioengineering (Basel). 2024 Dec 18;11(12):1287. doi: 10.3390/bioengineering11121287 (PMC11674002; doi:10.3390/bioengineering11121287)
Supplement: Supplementary file 1 [file bioengineering-11-01287-s001.zip › bioengineering-3306558-supplementary.pdf]

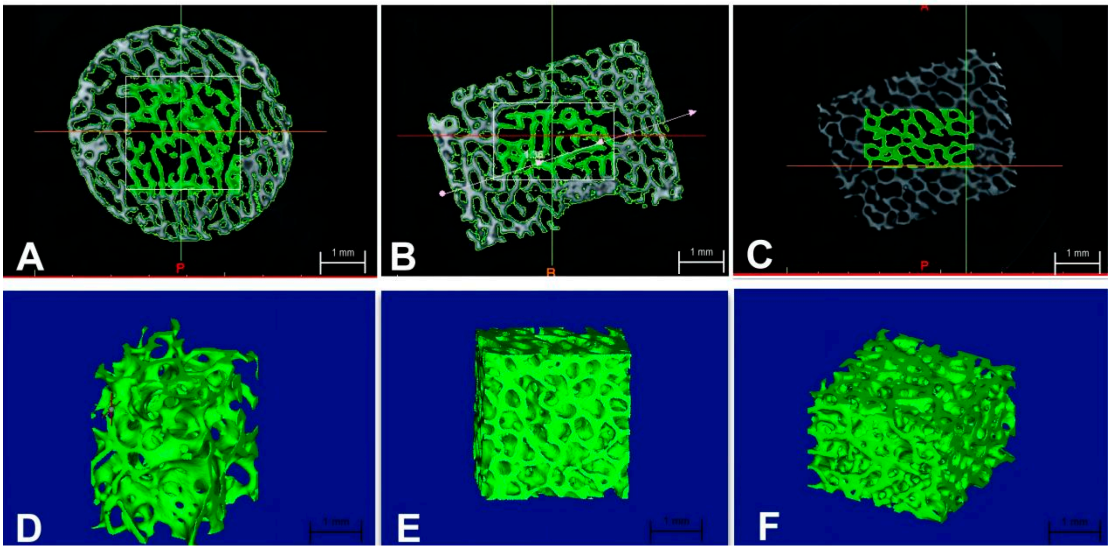

Figure S1. Porosity analysis on the three dimension reconstruction of representitive Micro-CT data of FPHA0.25 block.(A-C. standardized ROI selection; D-F. three dimension reconstruction of the selected ROI.).

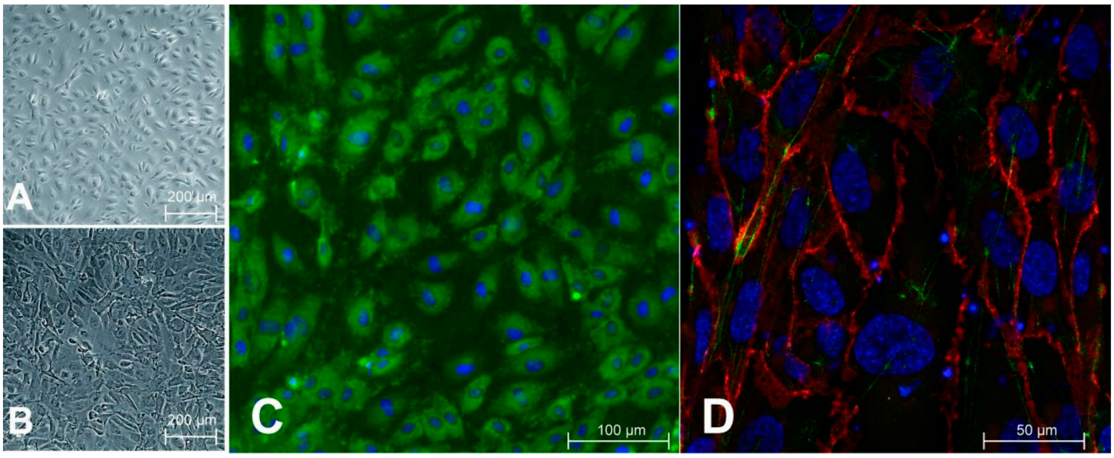

Figure S2. HUVECs were characterized by contrast microscope observation (A&B) and immunofluorescence staining of vWF (C, green) and VE-cadherin (D, red). The typical cobblestone shape of HUVECs observed under the contrast microscope and the positive staining of vWF and VE-cadherin indicated that the HUVECs used in this study were typical endothelial cells.

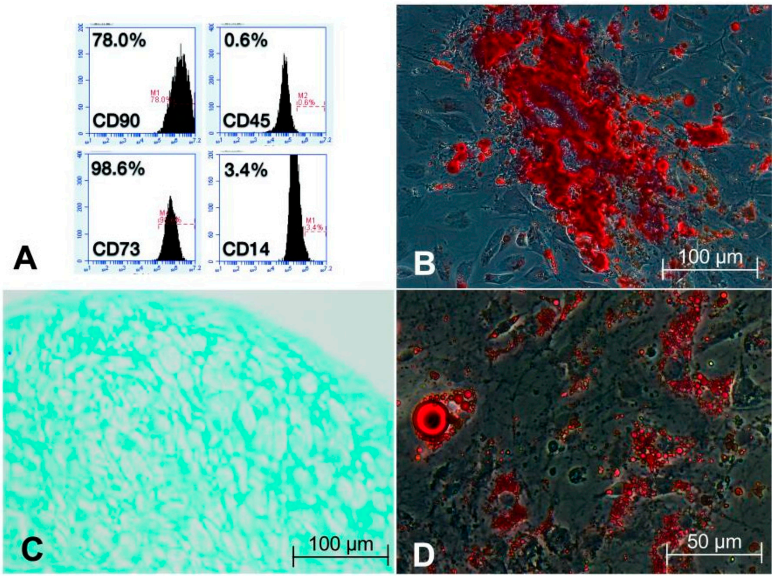

Figure S3. Multipotent differentiation potency and surface marker characterization and confirmation of HBMSCs: A. Flow cytometric analysis; B. Alizarin red S stain for osteogenic differentiation; C. Alizarin blue stain for chondrogenic differentiation; D. Oil-red O stain for adipogenic differentiation.

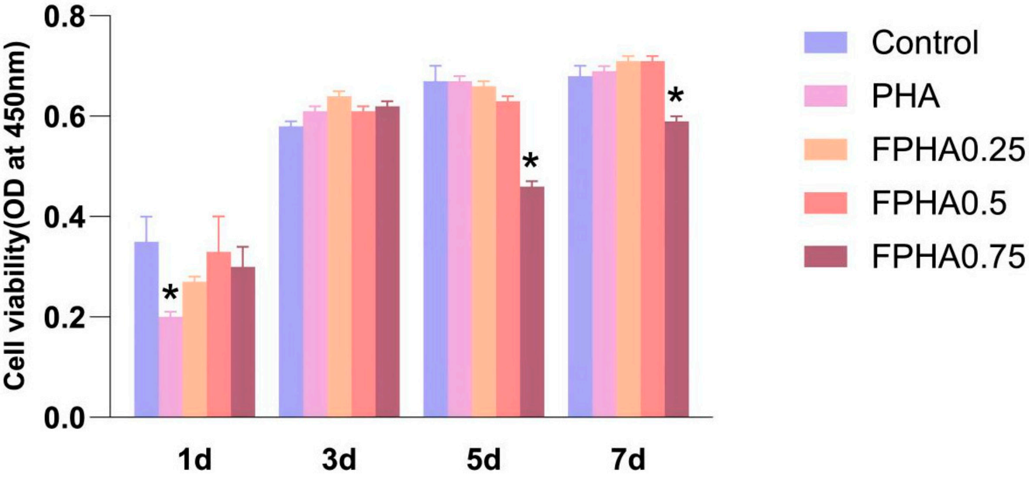

Figure S4. CCK-8 assay on the proliferation of HUVECs on days 1, 3, 5 and 7 cultured with 10% PHA and FPHA extracts. (* indicated significant difference comparison with the control, P<0.05).

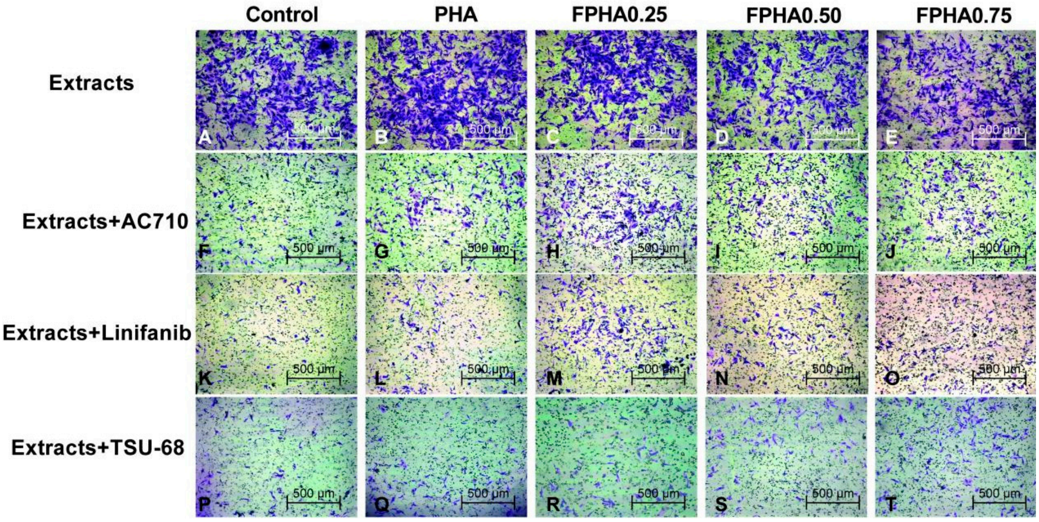

Figure S5. Representitive micrographs on the migration of HUVECs chemotacted by PHA/FPHA extracts at different concentration(6 h after addition of A-E. extracts only and F-J. AC710 (PDGFR phosphoration inhibitor); K-O. Limifanib (VEGF and PDGFR inhibitor); P-T.10 μM TSU-68) in the lower chamber (Cystal violet staining , ×50). Chemotactic effect of PHA/FPHA extracts at different concentration on the migration of HUVECs in vitro 6 h after addition of F-J. AC710(PDGFR inhibitor); K-O. Limifanib (VEGF and PDGFR inhibitor); P-T. 10ng/mL TSU-68 (KDR/PDGFR phosphoration inhibitor); * indicates P<0.05 compared to control.

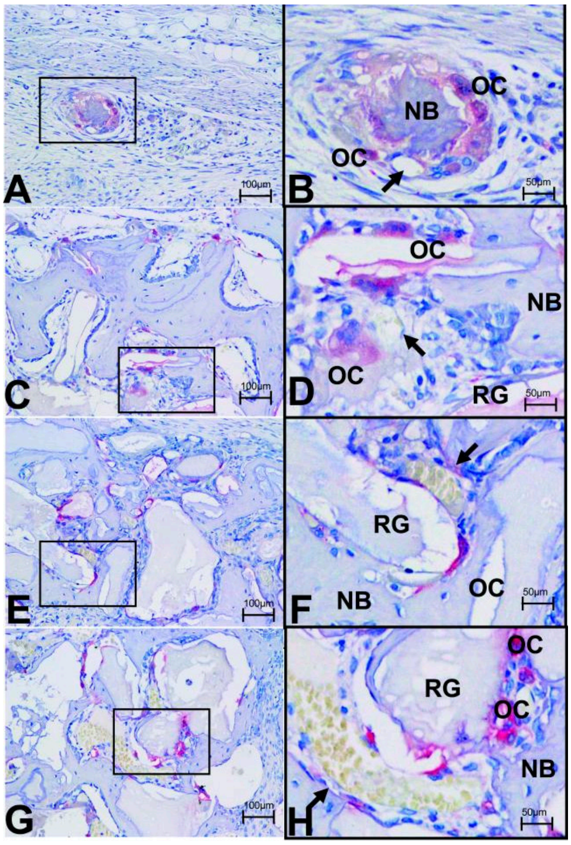

Figure S6. TRAP+ osteoclast resided surrounding the residual materials and microvessels 2 weeks postsugery. (A& B. blank control; C&D. PHA; E&F. FPHA0.25; G&H. FPHA0.50; figures in the right panel are enlarged close-up representative images area within the black frame of respective left images; OC. osteoclasts; RG. residual grafting materials; NB. new bone; black arrows. microvessles).
